# Evidence for an enterovirus as the cause of encephalitis lethargica

**DOI:** 10.1186/1471-2334-12-136

**Published:** 2012-06-20

**Authors:** Robert R Dourmashkin, Glynis Dunn, Victor Castano, Sherman A McCall

**Affiliations:** 1 Faculty of Life Sciences, London Metropolitan University, 166 Holloway Road, London, N7 8DB, UK; 2 Department of Virology, National Institute for Biological Standards and Control, Potters Bar, South Mimms Herts, EN6 3QG, UK; 3 Faculty of Computing, London Metropolitan University, 166 Holloway Road, London, N7 8DB, UK; 4 Department of Molecular Pathology, Armed Forces Institute of Pathology, 6825 16th St NW, Washington, DC, 20306-6000, USA

## Abstract

**Background:**

The epidemic of encephalitis lethargica (EL), called classical EL, was rampant throughout the world during 1917–1926, affecting half a million persons. The acute phase was lethal for many victims. Post-encephalitic parkinsonism (PEP) affected patients for decades. Our purpose was to investigate the cause of classical EL by studying the few available brain specimens. Cases of PEP and modern EL were also studied. Transmission electron microscopy (TEM) and immunohistochemistry were employed to examine brain from four classical EL cases, two modern EL cases and one PEP case.

**Methods:**

Standard methods for TEM, immunohistochemistry and RTPCR were applied.

**Results:**

27 nm virus-like particles (VLP) were observed in the cytoplasm and nuclei of midbrain neurons in all classical EL cases studied. Large (50 nm) VLP and 27 nm intranuclear VLP were observed in the modern EL cases and the PEP case. Influenza virus particles were not found. VLP were not observed in the control cases. TEM of cell cultures inoculated with coxsackievirus B4 and poliovirus revealed both small and large intranuclear virus particles and small cytoplasmic particles, similar to the VLP in EL neurons. In the EL brains, nascent VLP were embedded in putative virus factories and on endoplasmic reticulum (ER). The VLP in the cases of classical EL survived, whereas ribosomes underwent autolysis due to the lack of refrigeration and slow formaldehyde fixation of whole brain. The VLP were larger than ribosomes from well preserved brain. Immunohistochemistry of classical EL cases using anti-poliovirus and anti-coxsackievirus B polyclonal antibodies showed significant staining of cytoplasm and nuclei of neurons as well as microglia and neuropil. Purkinje cells were strongly stained.

A 97-bp RNA fragment of a unique virus was isolated from brain tissue from acute EL case #91558. Sequence analysis revealed up to 95% identity to multiple human Enteroviruses. Additional cases had Enterovirus positive reactions by real time PCR.

**Conclusions:**

The data presented here support the hypothesis that the VLP observed in EL tissue is an Enterovirus.

## Background

Encephalitis lethargica occurred suddenly in 1916–17 as an epidemic that was simultaneously reported in Vienna and in France, and continued until 1926, when it gradually disappeared. Reports of the disease spread throughout the world. A previous similar episode occurred in northern Italy in 1889–90 called *la nona.* Subsequent to the Italian outbreak, frequent reports of encephalitis in Europe were recorded (Hall AJ, Lumilean Lectures, Lancet 1923;**i:**731, quoted in [1].) During the course of the 1917–1926 epidemic, approximately half a million people world-wide were affected by EL. About one-third of the patients died acutely, one-third developed PEP and the remainder recovered almost completely. Sporadic cases diagnosed as EL have been reported up to the present time, but it has not been clear whether they share the same etiology as classical EL [2-4].

Dale, Church et al. [3], reporting on a series of modern EL cases, suggested that EL is an autoimmune disorder. They based this on clinical presentation, intrathecal oligoclonal bands (OCB), anti-streptolysin-O titers and autoantibodies reactive against human basal ganglia antigens in EL patients. Western blotting showed that 95% of EL patients had autoantibodies reactive against human basal ganglia antigens. While Dale et al. offer indirect evidence (see Discussion), our study presents direct evidence for a specific Enterovirus etiology for both classical and modern EL.

EL was thought to be infectious at the time of the epidemic. However, a controversy arose as to whether there was a direct etiological association with the contemporaneous “Spanish influenza” pandemic. Recently, McCall, Henry et al. [5] found no evidence for influenza RNA in brain from EL or PEP. Also, McCall, Vilensky et al. [6] found little historical data supporting influenza as the etiology of EL.

Anderson, Vilensky et al. [7] in a literature review reported on the neuropathology of 112 cases of acute encephalitis lethargica from the period of 1915 to 1940. They found that cortical damage was present in 75% of the cases, damage to the meninges and brainstem in half the cases, and the substantia nigra was damaged in 13% of the cases.

In the acute stage, EL presented as encephalitis with either somnolent or manic behavior, mask-like facies, muscular rigidity, involuntary eye movements, and a tremor which was distinct from the pill-rolling tremor of Parkinson’s disease. Importantly, some cases of PEP developed without a history of acute EL. Other PEP patients were somnolent for many years but recovered temporarily with L-dopa treatment introduced during the 1960’s. Sacks in “Awakenings” [8] documented the course of the patients’ therapy. The cause of EL has remained a mystery [1].

In routine autopsy procedure the whole brain is immersed in formalin, resulting in slow penetration and suboptimal preservation of ultrastructure. In the epidemic period, bodies were not refrigerated post-mortem. This was the practice even in the city hospital for the American capital as reported in the Washington Post, *Morgue Room at Gallingher Held Disgrace*, August 18, 1929. “The morgue at Gallingher Municipal Hospital is a disgrace to civilization…without any refrigeration system or other means of refrigerating bodies…the Gallingher morgue is worse than the most unmodern slaughter house in the country.” Hence brain tissue from the EL epidemic underwent autolysis to a greater degree than tissue obtained by modern autopsies. This had the effect of almost completely destroying the structure of neural ribosomes, whereas the VLP in the cases of EL were virtually intact.

In contrast to the fragile architecture of the normal neuron, Booss and Esiri [9] described virus particles in brain that were relatively well preserved in otherwise poorly preserved tissue.

This study seeks to define the cause of classical and modern EL. With the application of TEM, immunohistochemistry and molecular biology, evidence was obtained supporting the hypothesis that EL was caused by a member of the genus Enterovirus.

## Methods

### Brain tissue for TEM

The brain samples from human autopsy material were obtained from the pathology collections of the John Radcliffe Hospital, Oxford, UK; the Institute of Neurology, Queen Square, London UK, and from the former Armed Forces Institute of Pathology (AFIP), Washington DC, USA. These institutions approved the use of the tissue for research, and they are satisfied that no further ethical approval was required. In the case of the material from the UK, the principal author holds the “Release of Tissue Disclaimer” from the Thomas Willis Oxford Brain Collection, at the Neuropathology Department, John Radcliffe Hospital, Oxford UK.

In the case of the patient 98/1133, the data on the clinical history and pathological findings have already been published [2].

The following disease cases were examined by TEM:

AFIP:

Four cases of classical EL (#91558, #19940, #21225, #28065); one case of PEP (#132911), one case of Guamanian amyotrophic lateral sclerosis/parkinsonism/dementia complex (Guam disease, case #644255), one case of epidemic influenza (#1311056), and one case of coxsackie B4 encephalitis (#1064919).

Department of Neurology at John Radcliffe Hospital (JRH):

One case of multiple sclerosis (#31/3/03) and one case of modern EL (#98/1133).

Institute of Neurology, London:

One case of modern EL (#P31/06).

Normal control case examined by TEM from the period of the EL epidemic, from AFIP:

1. #8889. Diagnosis: Dural endothelioma with large cyst and pressure atrophy. Case from period of EL epidemic, date: 30/5/1919.

Normal modern control case examined by TEM; AFIP:

2. #AO2-18 Diagnosis: brain stem infarcts.

The Department of Neuropathology at JRH:

3. 06/126. Female 87 years. No cause of death known. No clinical brain disease known, little pathology.

4. 06/110 Male aged 70 years, diagnosis prostate cancer. No brain pathology.

5. 06/112 Female 87 years. Myocardial infarction and perforated gastric ulcer. No brain pathology.

6. 05/66. Female 26 years. Cystic fibrosis. No brain pathology.

7. 05/152. Female 51 years. Carcinoma of stomach. No brain pathology.

8. 196/08. Male 91 years. Prostate cancer, bronchropneumonia. No brain pathology.

Normal control case 8 (NP196/08) from JRH was collected directly at autopsy for TEM and fixed immediately in 3% glutaraldehyde in 0.1 M cacodylate buffer, for maximum preservation of structure.

### Method for TEM

Disease and normal tissues from human midbrain were dissected from paraffin embedded blocks to fragments 3 mm^3^ in size and cleared with Citroclear (HD Supplies, Aylesbury, UK) for 24 h. They were passaged though alcohol and PBS, then fixed in 3% glutaraldehyde in 0.1% cacodylate buffer, further dissected to 1 mm^3^, followed by 1% OsO_4_ in 1% cacodylate buffer. In one modern control case (196/08), a 3 mm^3^ fragment was excised from the inferior olive directly at autopsy and fixed with 3% glutaraldehyde followed by 1% OsO_4._ The tissue fragments after fixation were immersed in 0.5% uranyl acetate/water overnight at room temperature. They were then dehydrated and embedded in resin for TEM. Stained 0.5 μm sections of TEM blocks were used to choose appropriate blocks for the presence of abundant neurons. Ultrathin sections were cut, stained with 1% uranyl acetate and Reynold’s lead citrate, thinly coated with evaporated carbon for stability in the electron beam, and examined by TEM.

### Antibodies used in immunohistochemistry

The following were the primary antibodies used. The neutralizing titer of each antibody was reported by its institutional source.

1. Convalescent serum from human parvovirus infection (National Institute for Biological Standards and Control (NIBSC), Potters Bar, UK)

2. Mouse monoclonal antibody to human parvovirus B19 (Millipore, Watford UK)

3. Mouse monoclonal antibody to the conserved Enterovirus VP1 protein (clone 5 D8/1, Dako, Cambridge UK)

4. Mouse monoclonal antibody to Enterovirus type 71 (Dako).

5. Polyclonal rabbit antibodies to poliovirus types 1, 2 and 3 (NIBSC).

6. Polyclonal horse antibody to coxsackievirus B absorbed with human placental tissue (prepared by the Netherlands National Institute of Public Health and Environment (NNIPHE), Bilthoven, the Netherlands).

The anti-poliovirus antibodies for types 1, 2 and 3 were each diluted so as to be equivalent to 10 N.D. (neutralizing dose) /ml and then combined in equal volumes. The neutralizing titer of each polyclonal rabbit anti-poliovirus antibody was as follows: type 1, 1:2048; type 2, 1:256; type 3, 1:512. The anti-poliovirus antibody was reported only in relation to its neutralization titer, as the serum dilution for each virus type varied. The antibody pool was absorbed 5x for human brain antigens with a cleared and homogenized suspension of control human midbrain that had been fixed and embedded in paraffin for histopathology. The pool was used in the immunohistochemical tests at a starting dilution of 0.42 N.D., followed by two fold dilutions to 0.05 N.D. (Table 1).

**Table 1:** Immunohistochemical analysis using anti-poliovirus polyclonal antibody

**Neutralizing dose/ml**	**0.42**	**0.2**	**0.1**	**0.05**	**0 Antibody**
**Controls, UK**					
**06/112**	2+	2+	1+		
**06/110**	2+	2+	0		0
**06/126/7**	0				
**05/152-9**	2+	3+	0.5+		
**05/25-**	1+				0
**NP86/07**	0				
**05/47-11**	0.5				
**05/137/5**	0				
**05/66-28**	0				
**05/01-**	0.5				
**Controls, USA**					
**865183**	0				
**8889**	1+				
**8854127**	0.5+				
**883599**	3+		2+		0
**1311056**	1+				
**Guam 644255**	0.5			0	
**EL, USA**					
**21225**	2+	4+	2+	2+	0
**19940**	3+	3+	3+	2+	0
**91558**		4+	2+	1+	1+
**28065**	3+	1+	2+	0	0

Immunohistochemical analysis using anti-poliovirus types 1, 2 and 3 rabbit polyclonal antibody. Each type was diluted according to neutralizing titer to create equivalence of titer for the three polio types. The antibody was pooled and absorbed with human brain tissue (see Materials). Antibody quantitation is expressed as units of neutralizing dose (N. D.). Grades of staining intensity are expressed as follows: 0 – 0.5+ = no staining; 1+ = background intensity and not significant; 2+ − 4+ = significant staining of increasing intensity. Fractions of grades are the mean of more than one observation at the same experiment.

The antibody to coxsackievirus B (NNIPHE) neutralized a number of Enteroviruses to the following titers: Cox. B1: 20480; Cox B2: 20480; Cox B3: 10240; Cox B4: 10240; Cox B5: 2560; Cox B6: 1280. Cross-reactions: Enteroviruses E1, E8, E14, E27, E30 and CA9: 1:20, Enterovirus E20: 1:320. The antibody was used at a starting dilution of 1:3000, or 6.8 N. D. for the antibody titer to Cox B1 and Cox B2, followed by two-fold dilutions. The results were reported in terms of both the dilution titer and the neutralization dose for this antibody (Table 2). The neutralizing dose of the antibodies was not directly comparable to the antibody titers in the immunohistochemistry tests on brain tissue.

**Table 2:** Immunohistochemical analysis of control and EL brain using anti-coxsackievirus antibody

**Dilution**	**1:3,000**	**1:6,000**	**1:10,000**	**1:20,000**	**1:40,000**	**0 antibody**
**Neutralizing dose/ml**	**6.8**	**3.4**	**2**	**1**	**0.5**	**0**
**Controls, UK cases**						
**06/112**	1+		2+	2+	0	
**06/110**	2+	0				0
**06/126/7**	1+					
**05/152-9**	1+		0			
**05/25-**	1+					0
**05/47-11**	0					
**NP86/07-4**	0					
**05/137/5**	1+					
**05/66-28**	0					
**05-01-8**	0					
**Controls, USA**						
**865183-M**	1+	1+				
**8889C**	2+	0				
**8854127-13**	0					
**883599-11**	2+	2+	2+	2+		
**1311056-17**	1+					
**EL, USA Cases**						
**21225A**		3+	2+	2+		0
**21225B**		2+	3.5+	2+		
**19940C**	4+	0.2+	0			0
**19940B**		2.5+	4+	3+	0	0
**91558**	3+	2.5+	1+			1+
**28065B**	0	1+				0
**28065D**	3.5+	2.5+, 3+	3+, 3+	2+, 3+		0
**28065C**	0.5+	3+, 3+				
**28065A**	3+	2+	0			

Immunohistochemical analysis using anti-coxsackievirus B horse polyclonal antibody on brain from control and archival EL cases. The same expressions for quantitation are used as in Table 1. Case numbers followed by letters indicate different blocks from the same case. When tests were repeated, the two results are indicated in the same box.

### Tissue for immunohistochemical analysis

With the exception of control case NP 196/08, all specimens were formalin fixed, paraffin embedded autopsy brain. For immunohistochemistry, four cases of classical EL from AFIP whose clinical summaries are quoted below were studied, as well as 16 control specimens for the anti-poliovirus antibody, and 15 control specimens for the anti-coxsackievirus B antibody (Tables 1, 2).

### Method for immunohistochemistry

The immunohistochemical protocol and materials were supplied by Vector Laboratories (Peterborough, UK). The peroxidase-activated chromogen method for immunohistochemistry was preferred to immunofluorescence techniques because the brain sections examined by ultraviolet light microscopy showed autofluorescence that could not be quenched. In addition, the peroxidase-activated chromogen technique provided a permanent record of the experiments.

Brain sections were cleared of paraffin and passaged in industrial methylated spirits (IMS). Endogenous peroxidase was quenched with 3% H_2_O_2_ in methanol for 15 min. For antigen retrieval, slides were immersed in a steamer filled with 1 mM EDTA at pH 8.0 and were heated for 6 min at 98^o^C, then cooled for 6 min. The sections were then blocked with 5% normal serum for 1 h, using the same species serum as the secondary antibody. Biotin blocking was omitted because of the absence of biotin in human brain tissue (Esiri M., personal communication, JRH, Oxford UK), and as verified in our experiments (see Results: Immunohistochemistry). The sections were covered with the primary antibody diluted appropriately and incubated for 1 h at room temperature, then overnight at 4^o^C. Controls were performed by substituting PBS for the primary antibody. The sections were then exposed to the appropriate biotinylated secondary antibody, then treated with Vector ABC reagent (preformed avidin:biotin:enzyme complex). The slides were then rinsed and stained with Vector Nova Red. They were then cleared and mounted.

### Virus studies

One hundred μl of a 5 ml pool of 10^8^/ml infectious doses of the Sabin vaccine strain of poliovirus type 3 were inoculated into 25 ml flasks of L20B cells. This cell line was of murine origin that had been modified to express the human poliovirus receptor. The same dose of coxsackievirus B4 was inoculated into similar cultures of the Hep2C cell line, which is genetically identical to the HeLa cell line. Virus infected and control cell cultures were fixed with an equal volume of 3% buffered glutaraldehyde added to the cell culture medium for TEM at hourly intervals. The cells that had been released from the cell sheet following virus inoculation were collected by centrifugation of the supernatant medium and prepared for TEM as described below. The cells adherent to the flask were also collected and prepared for TEM.

## Results

### Analysis by TEM

A selection of TEM images is presented of control human brain, brain from classical EL, two modern EL cases and one case of PEP. They were not manipulated digitally to eliminate blemishes or for any other purpose. The original TEM negatives, photographic prints, and high resolution digital images may be viewed by contacting the first author. The magnifications quoted in Legends refer to the original photographic prints.

### TEM of control brain

**Figure 1:**
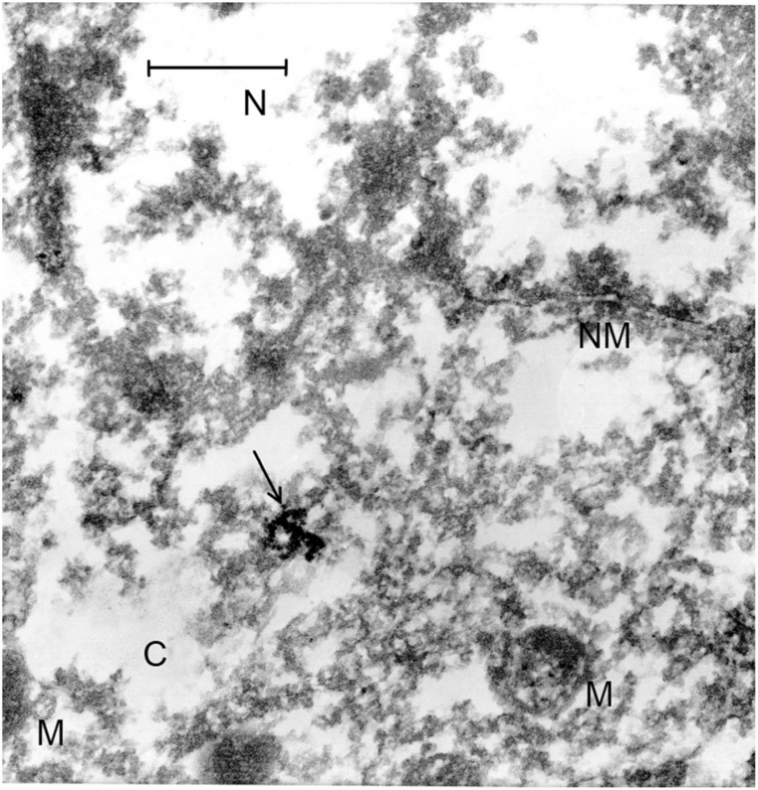
**(Mag. 66K).** Control case #8889, from the period of the EL epidemic. Diagnosis: dural epithelioma. An image is presented here of a neuron, demonstrating the severe degree of necrosis that was the result of the lack of refrigeration at that time. Other than severely damaged mitochondria (M), no cellular components are distinguishable. Arrow indicates artifact of TEM. **NM** = nuclear membrane, **N** = nucleus, **C** = cytoplasm **M** = mitochondria. Bar = 0.42 μm.

**Figure 2:**
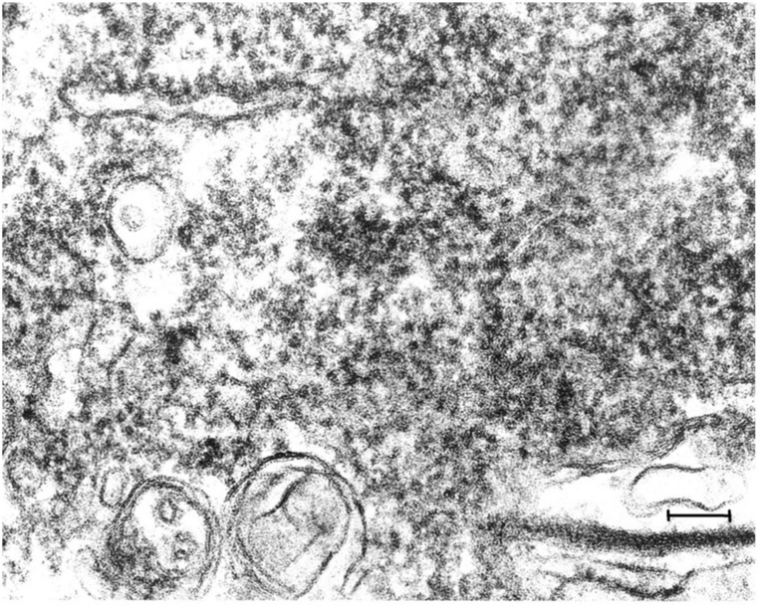
**(Mag. 163 K).** Modern control case NP196.08: ribosomes. On TEM examination of the brain that was optimally fixed for TEM at the time of autopsy (case 8), ribosomes of neurons were abundant, cell membranes and other cytoplasmic structures were intact. No particles similar to the VLP in the EL cases were observed in control brain either in the cytoplasm or in the nuclei. The contour of the ribosomes was irregular, in contrast with the images of the VLP in the EL cases, and their diameter was significantly less than the VLP illustrated in Figure 3. Bar = 100 nm.

As described above, the conditions in which autopsies were carried out during the EL epidemic resulted in severe autolysis of brain tissue, with the result that little of the normal ultrastructure of the neurons was preserved. This is illustrated in Figure 1, in which no evidence of the usual expression of cytoplasmic ribosomes in neurons can be discerned. In Figure 2, an image of a neuron from a modern autopsy case in which optimal preservation and fixation of tissue was observed, the morphology and frequency of neural ribosomes is illustrated. In the modern case controls, the quality of tissue preservation varied depending on the period of waiting time before body refrigeration was carried out and the method of brain fixation that was employed. No VLP were found in the control cases that were examined.

### TEM of EL Brain

**Figure 3:**
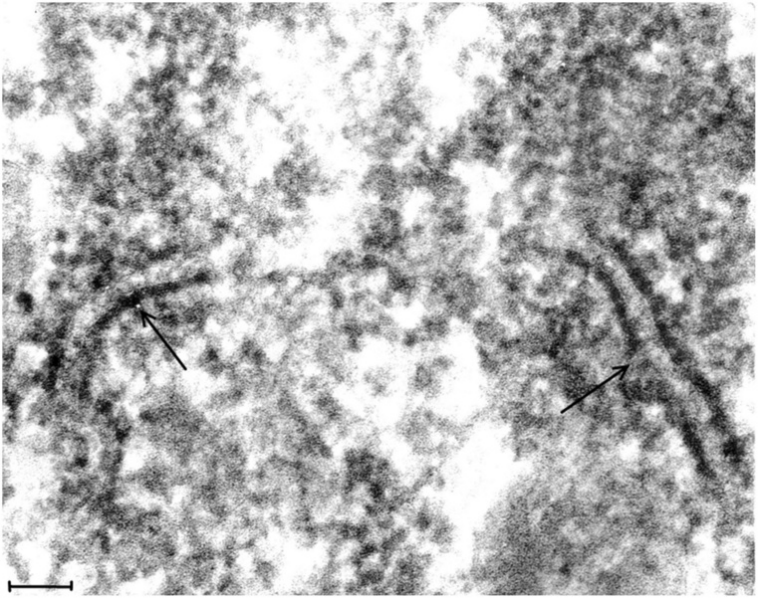
**(Mag. 165 K).** Classical EL case #21225. Cytoplasm of a neuron: VLP. This image illustrates the VLP in the cytoplasm of neurons in the EL cases. VLP were found only in the neurons of the cases of classical EL, modern EL, and PEP. A constant finding was the presence of ER membranes embedded in putative virus factories (arrows). The membranes were of varying length and were in association with small budding particles. VLP were also found embedded in putative virus factories. On comparing the cytoplasmic VLP in Figure 3 with ribosomes in a neuron in a modern normal brain in Figure 2, the VLP on visual inspection appear larger than the ribosomes. The difference on measurement of VLP and ribosomes is statistically significant (see Statistical Comparison of Particle Diameters). Additionally, the standard deviation results for the measurements of the VLP and normal ribosomes showed greater variation in the diameter of the VLP than that of ribosomes from modern control brain. This is consistent with measurements of VLP in various stages of assembly (see Figures 4, 5, 6, 7, 8, 9 and 10). Bar = 100 nm.

**Figure 4:**
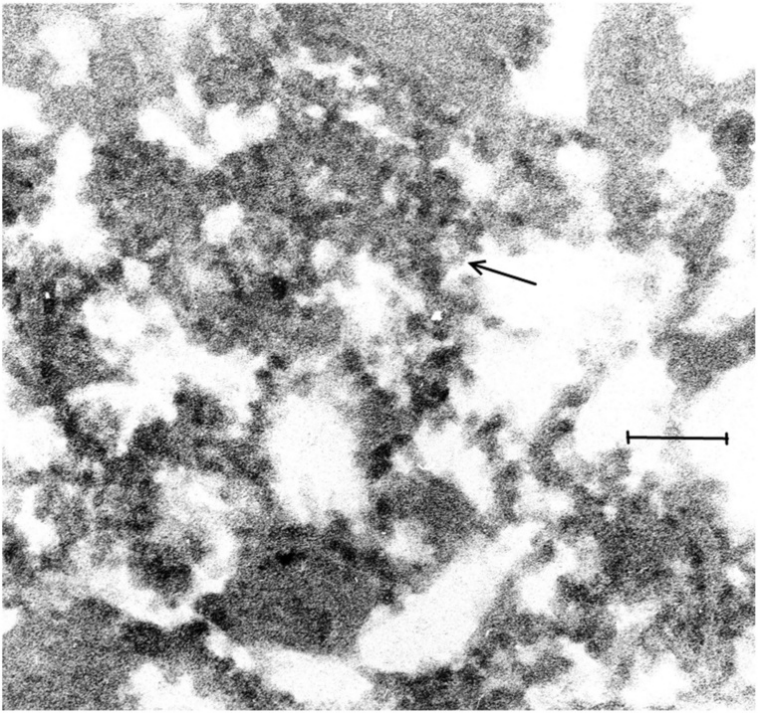
**(Mag. 150K).** EL case #19940. An image of VLP in the cytoplasm of a neuron. An image taken at low magnification (not shown) revealed large numbers of lipofuscin granules in the cytoplasm of a neuron, displacing the VLP to the periphery of the cell. Figure 4 is a high magnification of an area in the cytoplasm of the same cell, in which VLP are embedded in putative virus factory (arrow). Bar = 150 nm.

**Figure 5:**
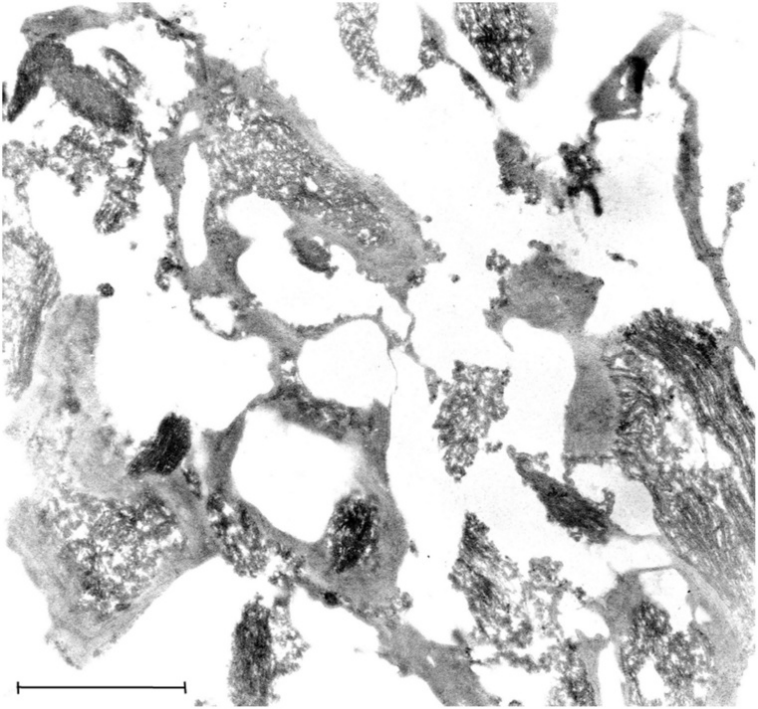
**(Mag. 26K).** EL case #28065B. This image illustrates a region of neuropil. There is extensive damage to the axons. There is swelling and obliteration of the myelin sheaths and dispersion of the nerve fibers. VLP were not observed in the capillary endothelium, neuropil, or cellular elements other than in the neurons. The TEM finding of involvement of the neuropil in EL is confirmed by the immunohistochemical staining of neuropil with anti-poliovirus antibody in EL tissue (see Figure 16). Bar = 1.2 μm.

**Figure 6:**
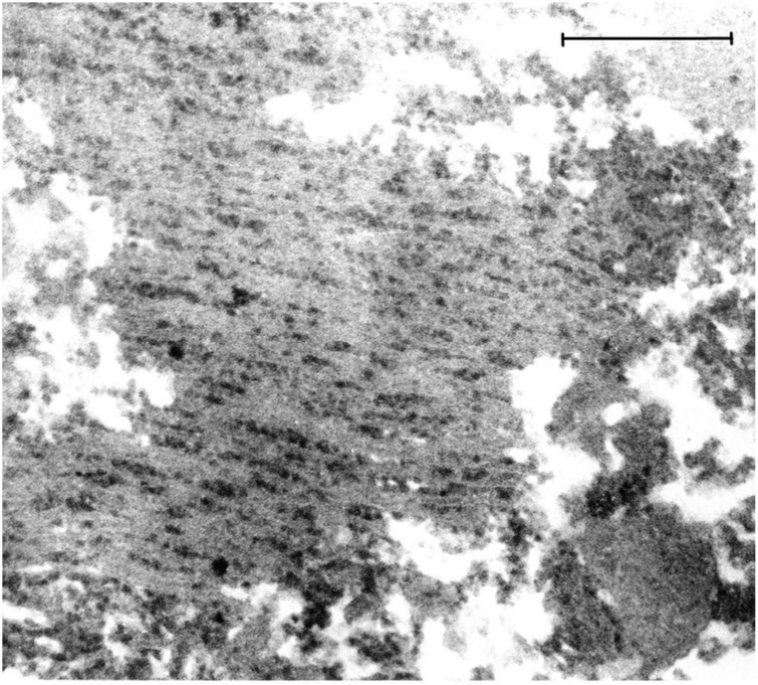
**EL case #19940 (Mag. 45K).** A large putative virus factory is illustrated in which an assembly of regularly arranged membranes is embedded. Between the membranes are large numbers of individual dense particles. The membranes are not aligned in a straight line, indicating that the image is not due to blade chatter. The particles bound to membranes are interpreted to be the precursors to the putative complete virus, i.e*.,* nascent VLP. Because of their high electron density and their size, which is smaller than that of the free particles, it is suggested that they consist of assemblies of nucleic acid without capsid protein. Similar membranes bearing VLP within putative virus factories were frequently observed in EL tissue. Bar = 1.2 μm.

**Figure 7:**
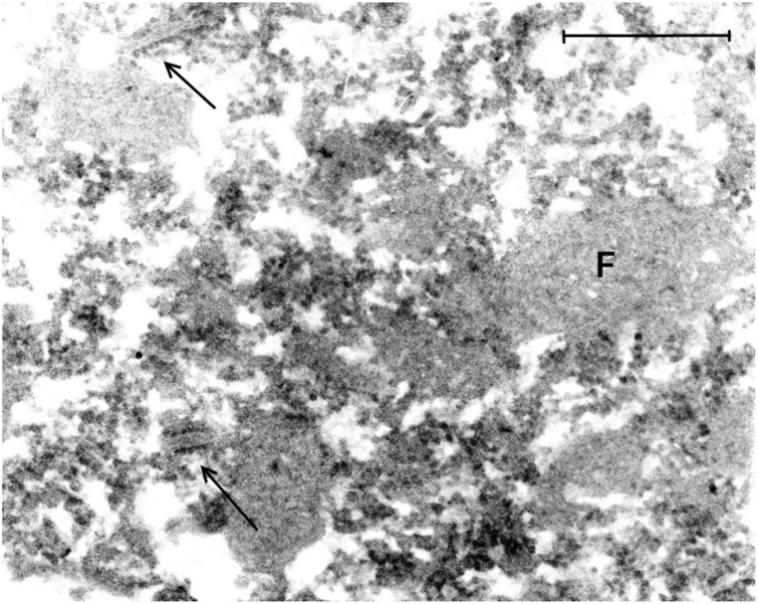
**(Mag. 45K).** Image from the same cell as in Figure 6**.** In this image of an adjacent area of the same cell as in Figure 6, the putative virus factory **(F)** is broken up into smaller fragments, with many nascent VLP embedded in the fragments and release of free VLP. There are a few short lengths of membranes with nascent VLP on their surface (arrow). Similar developmental stages of VLP were found in all the EL cases studied. Bar = 1 μm.

**Figure 8:**
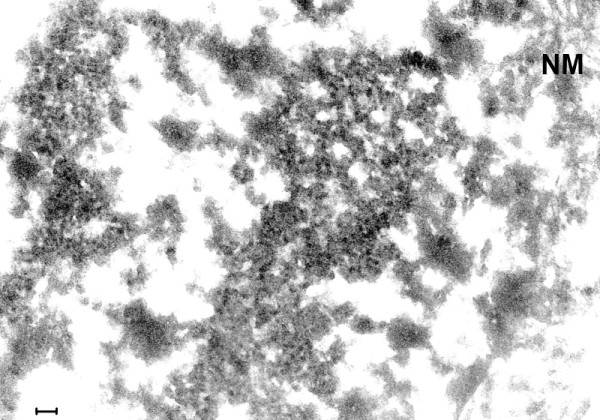
**(Mag. 71K) EL case #91558.** Area in cell nucleus. Large numbers of VLP are embedded in putative virus factories located in the cell nucleus. **NM** = nuclear membrane. The image of the nuclear membrane extends downwards along the right side of the image. Bar = 100 nm.

**Figure 9:**
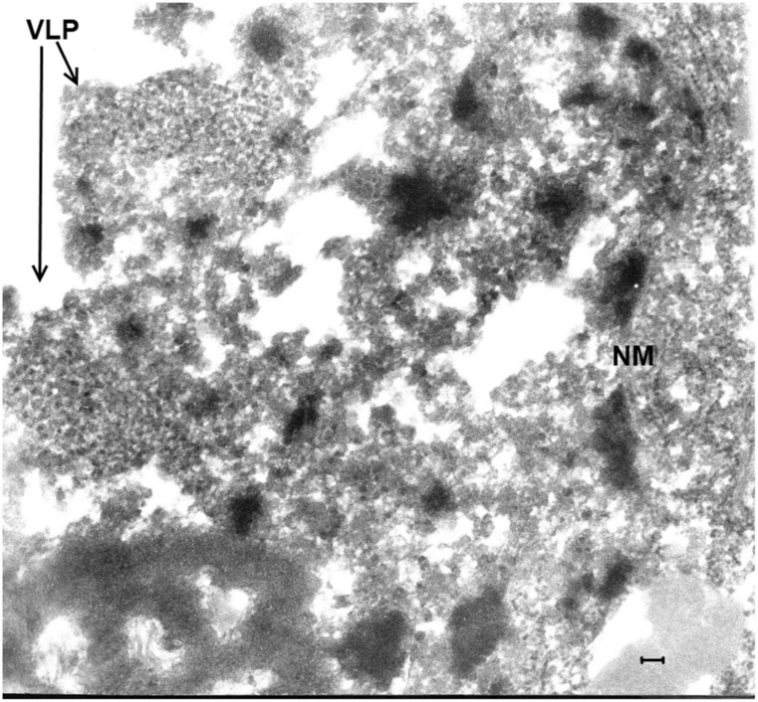
**(Mag. 48K).** EL case #28065. Area in cell nucleus. Putative virus factories with embedded VLP are indicated (arrows, **VLP**). “Empty” areas of cell are due to post-mortem damage. The nucleolus is shown in this image. **NM =** nuclear membrane. Bar = 100 nm.

**Figure 10:**
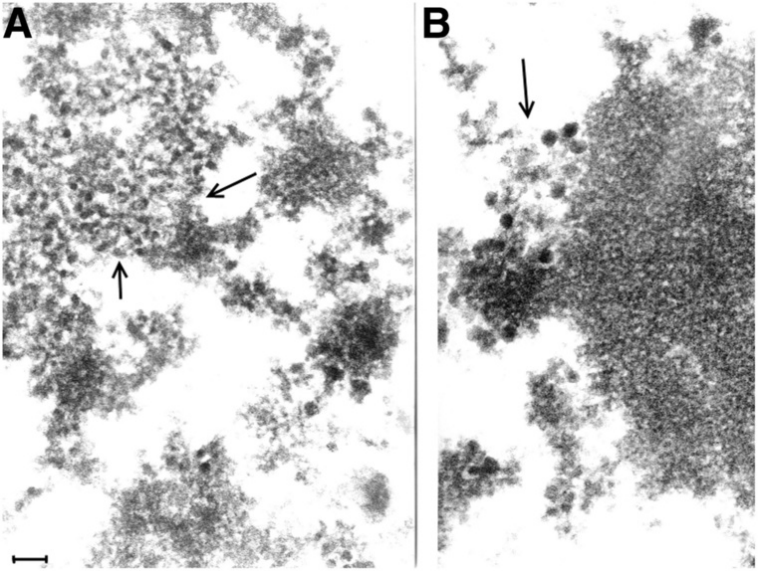
**A and 10B. (Mag. 76K). ** In the modern EL case P31/06, Figures 10A and 10B are selected areas from a TEM image of the nucleus of a neuron. In Figure 10A, a group of small intranuclear VLP (approximately 25 nm) is shown (arrow). In Figure 10B, close to and partly embedded in the nucleolus (arrow), there is a group of large (approximately 50 nm) intranuclear VLP. Bar = 100 nm.

**Figure 11:**
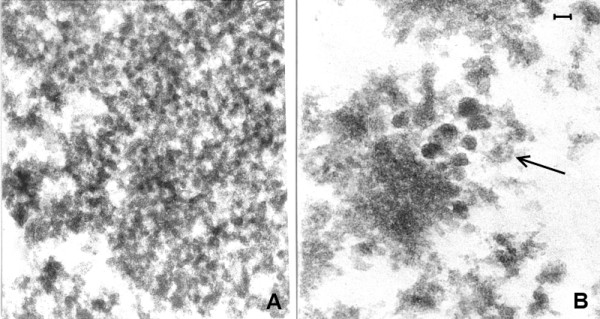
**A and 11B (Mag. 130K).** In the modern EL case 98/1133, two images are shown from two different neurons. Small cytoplasmic VLP (Figure 11A) and large intranuclear VLP **(L VLP,** arrow**)** (Figure 11B) are illustrated. Bar = 60 nm.

**Figure 12:**
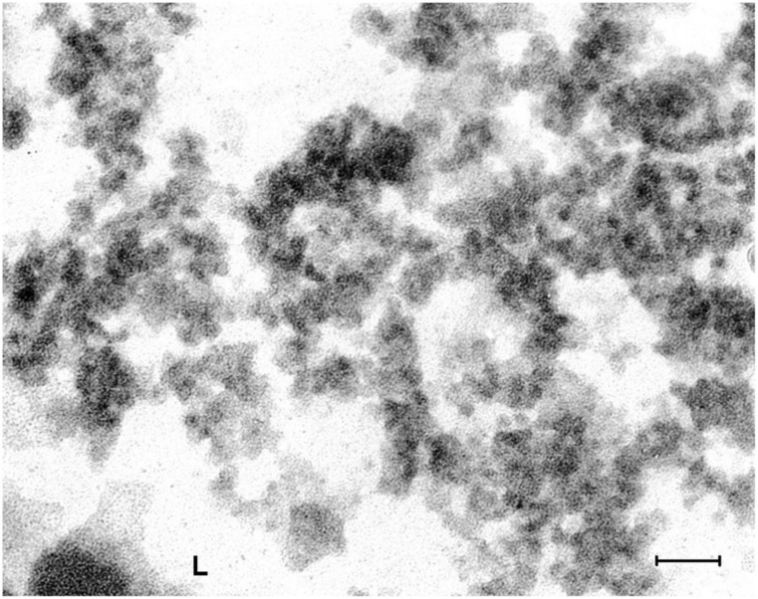
**(Mag. 175K). PEP case #1329111.** In an area of the cytoplasm of a neuron, there is a large number of VLP, each individually embedded in putative virus factory. **L** = lipofuscin body. Bar = 100 nm.

**Figure 13:**
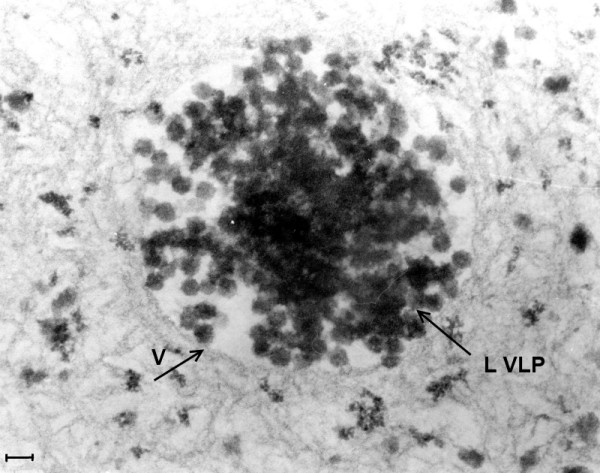
**(Mag. 100K).** PEP case #1329111. Figure 13 is in an area of the nucleus of another neuron. There is a cluster of many large (50 nm) VLP (**L VLP)** within a vacuole (**V**) situated in an intranuclear putative virus factory. The particles are of constant diameter, measuring approximately 50 nm, with geometrical silhouettes indicating icosahedral symmetry. There are also scattered individual dense particles, measuring larger than 50 nm, as well as clusters of small VLP in the same virus factory (not shown). Bar = 70 nm.

**Figure 14:**
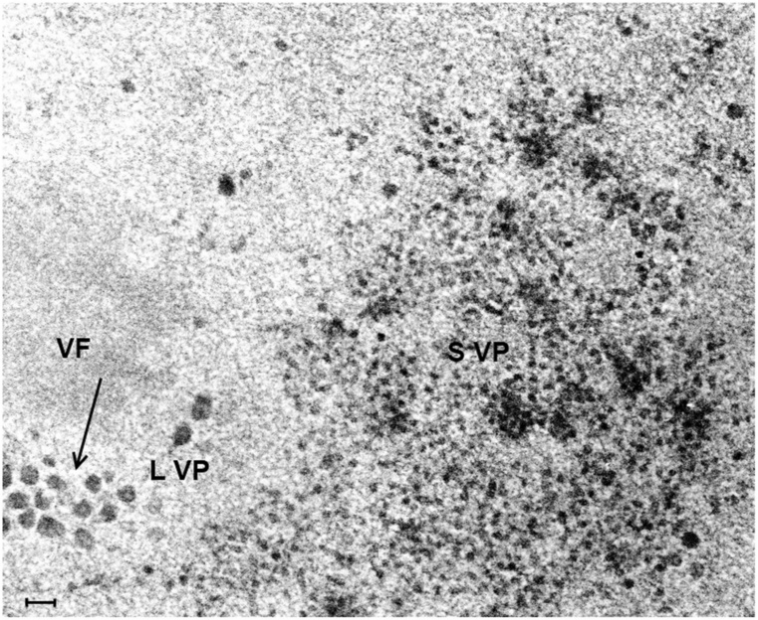
**(Mag. 74K).** Intranuclear replication of coxsackievirus B in cell culture. Figure 14 is a TEM image of a coxsackie B infected cell culture showing the intranuclear site of coxsackievirus B replication after 7 h incubation. Two types of particle are demonstrated in this TEM. Small particles are scattered throughout a virus factory, as well as occasional large particles. Large particles measuring approximately 50 nm are observed surrounding another virus factory. **SVP** = small virus particles. **LVP** = large virus particles, indicated by arrow. **VF =** virus factory. Bar = 100 nm.

**Figure 15:**
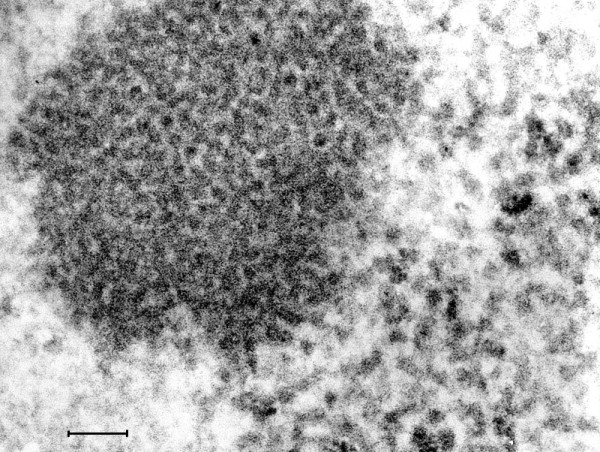
**(Mag. 216 K).** Intranuclear replication of poliovirus in cell culture. Figure 15 is a TEM image at high magnification of a 7 h cell culture infected with poliovirus, showing a cluster of intranuclear small virus particles. The clustered particles measure approximately 22 nm. Also there are larger particles of varying diameter measuring approximately 28 nm surrounding the cluster of small particles. There were no differences between the TEM images of coxsackievirus and poliovirus cultured *in vitro*. Bar = 100 nm.

Intranuclear and cytoplasmic small (27 nm) VLP were found in all cases of classical EL examined as well as in the case of PEP and the modern EL cases (Figures 3, 4, 5, 6, 7, 8, 9, 10, 11, 12). In EL case #91558 (Figure 8) and in EL case #28065 (Figure 9), small intranuclear VLP are demonstrated. In Figures 10A, 10B, 11A and 11B, which represent two cases of modern EL, both small and large (50 nm) intranuclear VLP were found. Figures 12 and 13 are from a case of PEP, in which small and large intranuclear VLP were detected. In Figure 13, a cluster of many large VLP was found, presumably in the process of replication. The finding of intranuclear VLP in EL and PEP brain tissue is in accordance with the TEM finding of intranuclear replication of Enterovirus particles *in vitro* (Figures 14, 15).

Retrospective examination of the classical EL cases (Additional file 1) did not reveal 50 nm VLP. No influenza virus particles were found in EL or PEP brain tissue by TEM.

### TEM of a case of coxsackie B4 virus encephalitis

Case #1064919. Brain neurons in this case showed many intranuclear small VLP, similar to those found in EL brain. Images are not shown, but are available on request.

### TEM of a case of human influenza

Case #1311056. Examination of brain tissue by TEM showed intracytoplasmic influenza virus particles taken up by a phagocytic cell. No particles were found resembling the VLP described in cases of EL. Images are not shown, but are available on request.

### Statistical comparison of particle diameters

Measurement of ribosomes from neurons of modern control human brain that was fixed under optimal conditions (case NP196/08) was carried out on a TEM paper print at 100,000x magnification. (Cf. Figures 2 &3, at higher magnification) Similar measurements of VLP in a neuron in the brain of EL patient #21225 were performed in the same way. Clearly defined ribosomes and VLP were chosen for measurement. The measurements in two dimensions of both ribosomes and VLP were determined using a 10x loupe. Mean and standard deviation (S. D.) and the T test were calculated from measurements of 40 VLP in each sample. The diameter of midbrain ribosomes: mean = 20.4 nm, S. D. = 3.35 nm. Diameter of cytoplasmic EL VLP: mean = 26.6 nm, S. D. = 4.43 nm. T test: p <0.01. In our discussion, the measurements are rounded to 27 nm for VLP, 20 nm for ribosomes.

The greater variation in size of the VLP as compared to ribosomes may be due to different stages of virus assembly. The VLP attached to membranes or embedded in putative virus factory were smaller than the free VLP.

### Immunohistochemistry: preliminary results

Preliminary TEM studies suggested that the small cytoplasmic VLP present in the neurons represented either a DNA virus (e. g., parvovirus) or an RNA virus (e. g., Enterovirus) as etiological candidates. Tests with Feulgen and DAPI stains for cytoplasmic DNA in cases of acute EL were negative. Employing immunohistochemistry, EL brain tissue was compared with controls using a monoclonal and a polyclonal antibody (human convalescent serum) to parvovirus B19, a DNA virus. For small RNA viruses in the Enterovirus group, a mouse monoclonal antibody specific to Enterovirus 71, and a mouse monoclonal antibody to the VP1 structural protein epitope in the genus Enterovirus (clone 5 D8/1) were used. The results from the monoclonal antibody experiments were not significant as there was crossreactivity with controls; absorption with control brain tissue would not be feasible with monoclonal antibodies as they are specific for one epitope. After exhaustive absorption with formaldehyde fixed human brain tissue the polyclonal antibodies were specific.

### Definitive results

In our preliminary experiments, formaldehyde sections of newborn mice genetically modified to express alpha synuclein (gift of Masliah E, UC San Diego, USA) were stained positively with the polyclonal anti-poliomyelitis antibody. However, the possibility that alpha synuclein in EL tissue may have cross-reacted with the antibodies used was negated by the finding of Josephs, Parisi et al. [10] that alpha synuclein was absent in PEP tissue. Had it been present in acute EL tissue, it would have persisted in the PEP patients’ tissue. However, the distribution of synuclein in neurons in brain carrying synuclein differs greatly from the antibody staining observed in our histochemical experiments.

The results of the immunohistochemical tests on brain tissue using a pool of rabbit polyclonal anti-poliovirus antibodies and a polyclonal horse anti-coxsackievirus B antibody described below are reported in Tables 1 and 2.

### Anti-poliovirus polyclonal antibodies

The polyclonal anti-poliovirus antibody pool described in Methods was used experimentally in serial dilutions. All four classical EL cases reacted positively with the anti-poliovirus antibody (2+ or greater); two cases to a dilution of 0.05 N.D./ml and two cases to a dilution of 0.1 N.D./ml. Removal of primary antibody in the immunohistochemical tests for EL and control brain tissue resulted in a zero signal. Omission of the biotin block step resulted also in a zero signal, demonstrating the absence of endogenous biotin in brain parenchyma. Of 16 control cases, one was positive to a dilution of 0.1 N.D./ml and three were positive to a dilution of 0.2 N.D./ml. The remaining 12 control cases were reported as 0, 0.5+ and 1+ at a dilution of 0.42 N.D./ml. These latter grades of reactivity were rated as below the threshold of significance. See Table 1.

**Figure 16:**
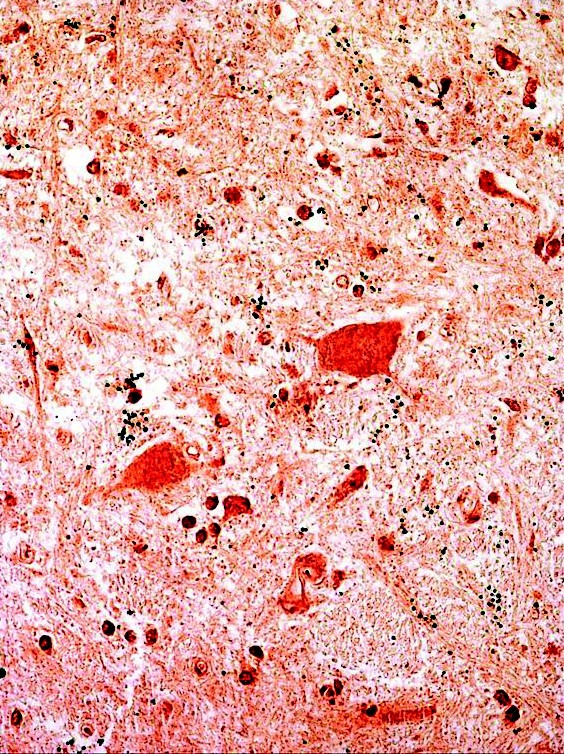
**Immunohistochemical staining of a midbrain area from EL case #21225.** A photomicrograph is shown at the greatest dilution of anti-poliovirus antibody at which the result was positive (0.05 N. D.). In this image, it may be noted that microglia and neuropil are stained as well as neurons. This is consistent with the TEM finding of extensive damage to the microglia and neuropil in the cases of acute EL (Figure 5). In areas of the tissue section in which the neurons were not stained, the surrounding neuropil and microglia were also unstained. Black formaldehyde pigment was present that was insolvent to 1% borohydride. Sections that were similarly prepared but with the omission of the primary antibody, showed no staining of neural, vascular or connective tissue elements, reflecting the absence of brain biotin. For highly sensitive immunohistochemical staining the sections were not counterstained and thus tissue structure was not visible in the control slides. The cytoplasm and nucleus of the neurons in the EL cases were positively stained to the same intensity, hence the nuclei were not clearly distinguished from the cytoplasm of the cells.

Figure 16 is an image of EL issue stained with the anti-poliomyelitis antibody at high dilution (0.05 N.D.).

### Anti-coxsackievirus B polyclonal antibody

On testing with the anti-coxsackievirus B antibody, three of four EL cases were found to be positive (rated as 2+ or more) to a dilution of 1:20,000 (1 N.D./ml.) Of 15 controls examined, two cases were positive to a dilution of 1:20,000. The rest, at an antibody dilution of 1:3,000 (6.8 N.D./ml), were rated as 0, 0.5+, or 1+, and were non-significant as stated above (Table 2). Two control specimens (06/112 and 883599) stained positively both with the anti-poliovirus antibody and the anti-coxsackievirus antibody.

### Molecular studies

McCall et al. [5] previously reported that influenza RNA was not detected in EL tissue. Based on the electron microscopic findings in EL of VLP that were similar to Enterovirus particles, consensus primers were designed for a highly conserved area in the 5′ untranslated region (5′-UTR) shared by several viral families. A 97-bp RNA fragment of a unique virus was isolated from acute EL case #91558 with the sequence: CTTGAGAGTCCTCCGGCCCCTGAATGCGGCTAATCCCAACCACGGAGCAGGTAATCGCAGCCCAGCGACCAGCCTGTCGTAATGCGCAAGTCTGTGG.

Sequence analysis reveals up to 95% identity to multiple human enteroviruses isolates (e.g. C and 99) and human coxsackievirus A24. The sequence has strong similarity to additional entero and coxsackie viruses, as well as several strains of human rhinoviruses (up to 93% identity), and human polio viruses (up to 93% identity). Additional samples had enterovirus positive reactions by real time PCR. These preliminary findings require further elucidation before publication of the molecular studies.

## Discussion

The hypothesis that EL is caused by an Enterovirus was explored using TEM, immunohistochemistry and RTPCR. Four cases from the 1917–1926 classical EL epidemic were studied. Preliminary TEM data indicated that small virus particles may be present. Possible small DNA viruses were the parvoviridae family [11,12] and viruses of the annellovirus genus, including Torque Teno virus (TTV) and MTTV [13,14]. Parvoviruses require actively dividing cells as fetal hematopoietic tissue as their source of DNA polymerase. Brain is therefore an unlikely site for the replication of parvoviruses [11]. Immunohistochemical findings utilizing anti-parvovirus antibodies did not support a parvovirus etiology. No clinical disease has been identified with the annellovirus genus, making it an unlikely candidate for EL [13,14]. These observations suggested that a small RNA virus such as Enterovirus is the most likely candidate.

Taxonomy of the Enteroviruses is as follows: Family – Picornaviridae. Genus – Enterovirus. Species – e.g. poliovirus or coxsackievirus. Enterovirus is a plus single-stranded RNA virus. The virion diameter has been reported to vary from 25 nm to 30 nm, depending on the virus species and preparation methods for TEM. Purified virus particles possess an icosahedral capsid coat consisting of 60 structural subunits, also referred to as capsomeres, with one copy of four capsid proteins VP1-VP4 in each subunit.

In our study of coxsackievirus B and poliovirus infected cell cultures, apoptosis of supernatant cells was complete at 4 h. This is consonant with the findings by other authors of apoptosis early in the viral replication of poliovirus [15] and coxsackievirus B infected cells [16]. We observed that virus replication in infected cell cultures took place in both the nuclei and cytoplasm. Different stages of virus replication were found without endoplasmic reticulum association detectable by TEM. Large clusters of electron dense, small (22 nm) virus particles were observed both in the nuclei and the cytoplasm of infected cells. Scattered throughout the virus factories were slightly larger virus particles measuring approximately 28 nm. In addition, large (50 nm) intranuclear particles were observed associated with virus factories. TEM of the control cell cultures showed no virus particles. The finding of intranuclear clusters of 22 nm particles surrounded by particles measuring approximately 28 nm suggests that incomplete 22 nm virus particles are first assembled in clusters; they subsequently develop into 28 nm complete virus particles that are then secreted into the cytoplasm and consequently to the extracellular space.

The finding of small (27 nm) and large (50 nm) particles in the same cell in the *in vitro* cultures that were similar in size to the two types of VLP described in cases of EL could be accounted for by a single infecting virus. The assembly of 50 nm particles may be the result of an alternative pathway for Enterovirus replication. Otherwise, there may be two distinct virus populations, one acting as a helper virus in association with Enterovirus for its expression. This would also apply to the finding of two types of particle in the modern EL cases and the case of PEP. This poses a subject for a future study of Enterovirus infected cell cultures. The presence of a helper virus would go toward explaining the sporadic epidemiology of EL. The possibility that the 50 nm particles in the *in vitro* virus infected cell cultures were an external contaminant is unlikely due a) to the TEM finding that the control cell cultures were free of virus particles; b) the seed virus was isolated by end point dilution; c) the 50 nm particles were also found in the modern EL brain tissues.

An experimental model for helper virus in Enterovirus infection was described by Cords and Holland [17,18]. In this study, synergy in virus replication was detected as the result of simultaneous infection in medium lacking in guanidine of cell cultures by two variants of polioviruses, one that was guanidine dependent, the other variant was guanidine independent. Replication of the guanidine dependent type was recovered by the concomitant replication of the independent type.

Being neurotropic, poliovirus and coxsackievirus B were the working hypotheses for the etiology of EL and therefore they were tested immunohistochemically [19]. The anti-poliovirus antibody staining was significantly positive to a high dilution with four classical EL specimens, and anti-coxsackievirus antibody staining was positive for three classical EL specimens, (Tables 1,2). In case #91558, which was rated as being negative for the anti-coxsackie antibody, the small sample of tissue available did not include neurons; the neuropil and microglia stained moderately. In positively stained EL specimens, the cytoplasm and nuclei stained equally. In specimens that included cerebellar tissue, Purkinje cells stained strongly. This was supported by the finding of a loss of Purkinje cells in the two modern EL cases reported by neuropathology examination, and also in the classic EL cases reviewed by Anderson, Vilensky et al. [7]. The involvement of the cerebellum in EL is consistent with the clinical tremor in EL patients.

The anti-poliovirus and anti-coxsackievirus polyclonal antibodies had been titrated against their respective viruses at their institutes of origin. High titers for these antibodies were found but cross-reactivity was reported (See Methods). Our findings suggest that the anti-viral specificity of the antibodies is related to the disease condition of EL. Two monoclonal antibodies against conserved Enterovirus structures were tested. The results were non-significant because of excessive cross-reactivity with control specimens. The manufacturers test their antibodies but do not guarantee absence of reactivity against human tissues. Because of antigen degradation in fixed autopsy material, the antibody titer for the immunohistochemical test could differ from the reported neutralizing dose.

VLP were not observed in the neuropil of brain tissue in areas affected by EL, which nevertheless stained positively with the polyclonal antibodies and which showed marked disruption by TEM. Therefore the immunogold technique using those antibodies would not discriminate more clearly between the VLP and other tissue components than did conventional immunohistochemistry and therefore was not performed.

In this report, a viral hypothesis is entertained. An alternative hypothesis of autoimmunity for the cause of EL, based on the work of “Dale, Church et al. [3] is described in Background. An editorial by Vincent [20] is paraphrased, which analyzed the autoimmunity hypothesis: - Dale identified autoantibodies by western blotting of soluble extracts of basal ganglia homogenate of human brain. The antibodies bound to several different polypeptide bands. Western blotting efficiently detects antibodies to non-conformational epitopes; it is likely to miss those potentially pathogenic antibodies that bind to conformational determinants. It would have been more informative to use a whole tissue or membrane preparation rather than a soluble extract. There are questions regarding the regional specificity since an analysis of different parts of the brain was not done. The term ‘antibasal ganglia antibody’ may be misleading. The presence of antibodies to different protein bands varying between the individual patients suggests they may be due to an immune mediated condition rather than causation. It is not yet clear whether the antibodies to the basal ganglia antigens” are crossreactive with streptococcus A antigens.

We deduce from our own data and that of Dale, Church et al. [3] that the autoimmune phenomena that they describe is secondary to an Enterovirus infection.

The electron microscopic, immunohistochemical and preliminary molecular findings make a prima-facie case for an Enterovirus etiology. Further study of the virology of Enteroviruses in cell culture is planned. TEM and immunohistochemistry of Enterovirus infected and control cell cultures will be carried out, physical purification of the two morphological forms of virus particles, and molecular biology of purified fractions of virus are projected.

The VLP found in EL tissue mirror the polio viral lifecycle *in vitro*[21,22]. Salonen, Ahola et al. [21] reported that on entry the viral genome migrates to specific perinuclear sites where the complementary minus strand RNA and plus strand RNA are synthesized. The authors present evidence that “poliovirus replication complexes consist of clusters of vesicles of 70–400 nm in diameter, which after isolation are large rosette-like structures of numerous vesicles interconnected with tubular extensions…The rosettes can dissociate reversibly into tubular vesicles, which carry poliovirus nonstructural proteins on their surface. In poliovirus infected cells a continuous proliferation and loss of ER membranes takes place”. Dales et al. [23] also found that polio replication is associated with ER membranes. In Dales’ TEM observations of poliovirus infected cells, single membrane bound bodies similar to the tubular vesicles that were reported by Salonen were described, commencing 3 h after infection and by 7 h overwhelming the remaining cell structure. In the late stages, complete virus particles, 26–28 nm in diameter, were observed within the tubules with an amorphous matrix. The complete particles were finally assembled in crystalline arrays in the cytoplasm. Dales et al. also observed ‘viroplasm’ in the cytoplasm of cells 3 h after infection, and aggregates of ‘granules’ 17–25 nm in diameter in the cytoplasm, unassociated with membranes, which they assumed to be nascent virus particles. Their micrographs also illustrate large numbers of individual nascent cytoplasmic virus particles outside the aggregates. Dales did not report the intranuclear replication of poliovirus.

However, the importance of the cell nucleus in poliovirus replication is demonstrable. Bienz et al. [24] described the migration of poliovirus proteins into the host cell nucleus. Kawanishi [25] and Anzai and Ozaki [26] infected FL cells (derived from human amnion) with poliovirus at 28°C for 20 h and found intranuclear crystals of assembled poliovirus capsids without cores. Follett, Pringle et al. [27] showed that a number of RNA viruses, including poliovirus, yielded much less virus when enucleated cells were infected.

Lipofuscin bodies are often found in large numbers in neurons of EL brain. They stain acid fast with carbolfuscin and positively with PAS, indicating their origin or association with lysosomes. They are frequently found normally in brain from the age of 9 to patients late in life in increasing frequency and numbers [28,29]. They are not specific indicators of viral infection. However, the large number of lipofuscin bodies found in the neurons of EL cases was abnormal for patients in the young age group that was typical for classical EL. They were also observed by light microscopy in the histopathological analysis of EL case #91558. Presumably they develop as a cellular reaction to virus pathogenesis.

During the EL epidemic, *in vivo* experiments were carried out supporting the hypothesis that EL was caused by a virus related to poliovirus. In 1921, Neustaedter, Larkin et al. [30] injected the brains of five macaque monkeys with a suspension of brain from a monkey killed by poliomyelitis. The polio infected brain suspension had been incubated with either convalescent serum from surviving EL patients, or with normal human serum or saline as controls. Monkeys injected with polio infected brain that had been incubated with EL serum were protected, whereas controls either died or were paralyzed. This result suggests that antibodies from EL patients neutralized poliovirus due to antigens common to poliovirus and the EL virus. Neustaedter’s experiment was similar to experiments a decade earlier demonstrating the neutralization of poliovirus by convalescent sera from polio patients. There is no reason to suppose that contemporary technical limitations invalidate this classical experiment.

More recently, persistent coxsackievirus B encephalitis was reported in both immunodeficient [31] and immunocompetent patients [32]. The observations that coxsackievirus B infection may persist in the central nervous system are significant for post-encephalitic parkinsonism, a chronic, presumably infectious syndrome. Evidence for chronic Enterovirus infection has been reported in the post-viral fatigue syndrome [33,34]. Their observations are consonant with our finding VLP in brain in a case of PEP (see Figures 13,14).

The rhinoviruses are members of the genus Enterovirus. They are the most frequent cause of the common cold. Infection in children may result in bronchiolitis and asthma. However, there are no reports of central nervous system involvement by rhinoviruses. It is unlikely, therefore, that they may be implicated in the etiology of EL. The rhinovirus capsid is icosahedral in structure, with a diameter of approximately 30 nm [35].

As part of the control study, TEM of brain from a patient with fatal H3N2 influenza was carried out. Intracellular influenza virus particles were found, presumably following an agonal surge of infection and breakdown of the blood brain barrier. No particles resembling VLP were present. This observation demonstrates that influenza virus particles, were they present, may be readily recognized by TEM in autopsy tissue of human brain.

## Conclusions

This study is concordant with the conclusions of McCall et al. [5,6] contesting the influenza theory of EL. Evidence is presented favoring an Enterovirus etiology of classical EL. It is supported by the TEM finding of small VLP in the cytoplasm and nucleus of neurons from classical and modern cases of EL and one case of PEP. The VLP were differentiated from ribosomes by the following findings. 1) The developing VLP were either in putative virus factories or nascent VLP particles attached to ER membranes. 2) There was nearly complete loss of ribosomes in normal control brain from the EL epidemic period in contrast to the predominant preservation of VLP in the cases of classical EL. 3) The diameter of the complete VLP observed by TEM was significantly greater (26.6 nm) and more variable than ribosomes in neurons from normal control brain (20.4 nm).

In two cases of modern EL and in one case of PEP examined, 50 nm intranuclear particles were found in addition to the small VLP. Similarly, in poliovirus and coxsackievirus B infected cell cultures, intranuclear aggregates were observed of 22 nm, 28 nm and 50 nm virus particles. The Enterovirus hypothesis is further supported by positive immunohistochemistry results employing polyclonal anti-poliovirus and anti-coxsackievirus antibodies on EL brain tissue.

Together with the TEM and immunohistochemical findings, preliminary RTPCR leading to sequence data of a 97-bp RNA fragment isolated from one case of epidemic EL strongly point to the genus Enterovirus as the cause of EL. Further information on the authors is available in Additional file 2. 

### Consent document

Written informed consent was obtained from the relatives of the case P31/06 for publication of the patient’s clinical history and pathological findings. A copy of the written consent is available for review by the Editor-in-Chief of this journal.

## Competing interests

In publishing this work, there are no competing interests for any of the authors.

## Authors’ contributions

RD developed the plan of research, carried out the electron microscopy studies including all the technical procedures, and also carried out all the procedures for immunohistochemistry. RD also developed the interpretation of the results and wrote the major part of this paper. SM supplied from AFIP the clinical material of archival encephalitis lethargica and controls from the epidemic period, as well as the clinical and pathological histories and the initial molecular data for this study. With unique insight, he gave excellent scientific advice and suggestions all during the course of the study, edited the drafts of the paper and contributed to the text and the interpretation of the results. GD provided *in vitro* cultures of Enteroviruses for electron microscopic study, as well as the antibodies for immunohistochemistry from NIBSC. VC converted the photographic images presented in this study to digital format for maximum resolution. All authors read and approved the final manuscript.

## Funding

The Sophie Cameron Trust, 107 St Thomas Road, Trowbridge, Wilts., BA 14 7LT. Email: Mail@sophiecamerontrust.org.uk The trust funded essential consumable supplies for Dr Dourmashkin’s research and contributed to the cost of publication.

## Pre-publication history

The pre-publication history for this paper can be accessed here:

http://www.biomedcentral.com/1471-2334/12/136/prepub

## Supplementary Material

Additional file 1 Patient Case Histories and Neuropathologic Findings: Classical EL cases. Click here for file

Additional file 2 Authors’ information. Click here for file
