# Supplementary material for: Evidence for an enterovirus as the cause of encephalitis lethargica
Source: BMC Infect Dis. 2012 Jun 20;12:136. doi: 10.1186/1471-2334-12-136 (PMC3448500; doi:10.1186/1471-2334-12-136)
Supplement: Additional file 1 — Patient Case Histories and Neuropathologic Findings: Classical EL cases. [file 1471-2334-12-136-S1.doc]

**Classical EL cases**

The following classical EL case histories were obtained from AFIP and were dated between 1923 and 1927, inclusive.

Classical EL case #19940. A 39-year-old man whose symptoms began with pain, abdominal hyper-reflexia and spasm. He had tachycardia, dry mouth and temperature of 37.7^o^ C. He was restless and was in constant motion. He twitched spasmodically, had muttering speech, a coarse intentional and resting tremor, unsteady gait and fell backward to the left. From the 15^th^ to the 22^nd^ day of illness, his abdominal reflexes dissipated but other reflexes were normal. He experienced bouts of irrationality. He was not aware of his behavior, but answered questions sensibly. One month onward, he began having urinary retention with as much as 1330 ml on catheterization, but urinalysis was normal. Thirty-four days later, he was stuporous and died with an axillary temperature of 41.7^o^C. His blood leukocyte count was 7,200 with a differential of 30 lymphocytes, 4 monocytes, 63 neutrophils and 4 bands. His Wassermann reaction was negative at autopsy. The cause of death was attributed to EL, myoclonic type.

*Pathology report:*

The parenchyma of the pons contains multiple foci of dense, perivascular round cell infiltrates within the Virchow-Robin spaces of small vessels, consisting predominantly of lymphocytes. Similar perivascular infiltrates involve the region of the locus ceruleus, although there is no evidence of acute neuronal injury. Random hyaline globules are noted within the cytoplasm of the abducens and facial nuclei. Sections of medulla and spinal cord are characterized by similar inflammatory changes including scattered foci of leptomeningeal round cell infiltrates with superficial extension into the Virchow-Robin space. There is marked vascular congestion with occasional small, perivascular ring hemorrhages. Inclusion bodies, including intraneuronal intranuclear Cowdry A variants suggestive of viral disease and intracytoplasmic Lewy bodies are not present in these sections or in the other acute cases.

Classical EL case #91558. This was a case of EL that took place during the 1917-25 epidemic. The tissue blocks were submitted in 1943 at the request of the AFIP from Jefferson Medical College, Philadelphia. The exact date and history of the case is unknown.

*Pathology report:*

The dorsal portion of the rostral brainstem is available for review. There is an intense perivascular inflammatory response within the parenchyma, consisting of densely distributed perivascular round cell infiltration in the Virchow-Robin spaces with a preponderance of lymphocytes and admixed plasma cells. There are similar inflammatory cells within the parenchymal stroma with evidence of tissue injury ranging from rarifaction and gliosis to frank necrosis of white matter with macrophage activity. Neurophagia is present within the adjacent gray matter. Congestion with small, perivascular ring hemorrhages is observed. This case is histopathologically most typical of an acute EL and reflects a more severe degree of parenchymal injury than cases #19940 or #28065.

Classical EL case #28065. This was a 19 year old man who became dizzy and had a seizure without fever. In two days dizziness returned with right temporal headache and no focal findings. Four days later, he was stuporous but could be roused, with slight nuchal rigidity and an equivocal Babinski, right hemiparesis and sluggish reflexes throughout. He was normotensive and had a temperature of 38.9^o^ C. The leukocyte count was 13,000 with 75% PMNs, falling in succeeding days to 6,800 with a negative blood culture. The CSF was clear, had 48 cells per μl, negative globulin, sugar of 85.4 mg/ml, negative Wassermann and colloidal gold. His eyes did not converge, but he had intact corneal reflexes and no ptosis. On the left side there was anesthesia and areflexia, and on the right side an equivocal Babinski, hyper-reflexia, and pseudoclonus on passive motion. Abdominal, epigastric, cremasteric and left patellar reflexes were absent. On June 13 he died with tachycardia and fever of 41.7^o^C. There had been no polio or influenza outbreak in his environment for two years. His medical and trauma history was negative.

*Pathology report:*

The histopathologic changes are similar in all sections, but varied in degree. Sections of neocortex contain occasional leptomeningeal round cell infiltrates, consisting predominantly of lymphocytes with admixed plasma cells. The parenchyma contains occasional glial nodules in the absence of significant perivascular round cell infiltrates. The gyral white matter is rarified with associated gliosis and scattered perivascular round cell infiltrates composed predominantly of lymphocytes. There is prominent congestion in these sections with no evidence of perivascular ring hemorrhages. The section of pons contains minimal focal leptomeningeal round cell infiltrates with occasional glial nodules and prominent perivascular round cell infiltrates, consisting predominantly of lymphocytes. There is severe vascular congestion with scattered perivascular ring hemorrhages involving small, thin-walled vessels. The sections of medulla contains scattered foci of leptomeningeal round cells and random foci of perivascular round cell infiltrates, composed predominantly of lymphocytes with admixed plasma cells.

Classical EL case #21225. A 43 year-old man who became ill on with “lethargic encephalitis.” No further clinical details were available.

*Pathology report:*

Grossly the brain was unremarkable, but had diffuse lymphocytic infiltrate in caudate, choroid plexus, medulla and cord most marked around the fourth ventricle. Ganglion cells in medulla showed rather advanced chromatolysis of individual cells, with atrophy and loss of nuclear staining. There were numerous hemorrhages from small and medium-sized vessels throughout the section of the medulla with edema most marked near the ventricle floor.

**Modern EL cases**

Modern case P31/06. The patient had no significant previous medical history and only a vague history of autoimmune disease in her family. At the age of 18 she developed a flu-like illness with vomiting and headache. Over the days she developed behavioral changes (such as disinhibition and aggressive behavior), hallucination, feelings of derealization and episodes of “freezing” of her mobility. She also complained of pain in the back when moving the neck.

She was admitted to a hospital urgently and had a CT scan of the brain which showed no abnormality. A spinal fluid analysis suggested encephalitis and Acyclovir was started. During the next days she developed neck dystonia and possibly oculogyric crisis, agitation and perseveration, later presenting double incontinence and mutism. She had a seizure and later a cardiac arrest due to septicemia. A following MRI of the brain revealed numerous anoxic lesions. She remained in intensive care for 3 months in an unresponsive state.

She was discharged to home bedridden and severely spastic; communication was reduced to looking at care personnel, although she seemed to understand some verbal commands and would often calm down with music. During the next years her condition had no significant changes and she received palliative care. A brief trial of Levodopa to a maximum of 125 mg a day did not result in significant benefit and this medicine was not tried again. After a failed trial of Tizanidine the spasticity was treated with Botulinum toxin injections with some benefit. At the age of 24, while having physiotherapy, she suddenly became pale, and 3 hours later “chesty and wheezy”, and went into cardiac/respiratory arrest. She died of a chest infection with a diagnosis of encephalitis lethargica and possibly anoxic brain damage.

*Pathology report: Gross observations (Royal United Hospital, Bath).*

300 ml pleural effusion was noted. The bronchi were congested and filled with mucopurulent exudate. The lungs showed bilateral bronchopneumonia. The brain showed diffuse atrophy consistent with EL.

*Microscopic observations, (Institute of Neurology, London).*

Histological slides were stained with routine methods including haematoxylin and eosin and luxol fast blue-cresyl violet and were used for immunohistochemistry with antibodies to CD68, glial fibrillary acidic protein (GFAP), tau (AT8 antibody) and ubiquitin.

Histological slides show that the leptomeninges are focally thickened. In the anterior frontal cortex there is neuronal cell loss and astrogliosis in a pseudolaminar distribution and this process is most severe in the 3^rd^ cortical layer. Such changes are often more pronounced in the depths of the gyri than over the gyral crests. The neuronal cell loss accompanied by gliosis and an increase in number of microglia is much more severe in the posterior frontal, parietal and occipital cortices where there is also cortical necrosis with preservation of the molecular layer. Such changes are less severe in the temporal cortex. No microglial nodules are found in any of the cortical areas investigated. In the most affected regions the white matter shows severe rarefaction with myelin pallor, axonal loss and gliosis. A few blood vessels surrounded by sparse mononuclear inflammatory cuffs are noted. The inflammatory infiltrates also include pigment-laden (lipofuscin) macrophages. There is near complete loss of neurons accompanied by severe gliosis in the CA1 hippocampal subregion. The CA4 hippocampal subregion is also affected by nerve cell loss and astrogliosis, but the changes are less severe here than in the CA1 subregion.

There is significant degree of neuronal cell loss accompanied by astrogliosis in the striatum. These changes are the most conspicuous in the dorsal aspect of the caudate nucleus and putamen. The globus pallidus is affected by astrogliosis. The dorsal aspects of both thalami containing lateral and anterior thalamic nuclei show severe neuronal loss and gliosis. The mammillary bodies show rarefaction of the neuropil and significant degree of astrogliosis.

The neuronal cell population of the substantia nigra is well preserved with minimal pigment incontinence. There are neither Lewy bodies nor neurofibrillary tangles in the nigra. The middle one third of both cerebral peduncles, corresponding to the corticospinal tracts, shows significant atrophy, myelin pallor, astrogliosis and an increase in number of microglial cells/macrophages. There is severe loss of neurons in the inferior olive. The cerebellar cortex shows significant degree of atrophy with near complete loss of Purkinje cells and severe depletion of the granule cells. The cerebellar cortex is affected by significant degree of astrogliosis. The cerebellar white matter shows severe pallor and gliosis. There is severe nerve cell loss and astrogliosis in the cerebellar dentate nucleus. A small number of axonal swellings are found in the dentate.

The major neuropathological findings are those of extensive and severe nerve cell loss and gliosis in cerebral cortex, basal ganglia, brainstem and cerebellum. The pattern of the nerve cell loss corresponds to that seen after severe global cerebral hypoxia. There are only scattered small foci of chronic inflammatory cells and no conclusive microscopic features of an encephalitic illness are seen. It is of note, however, that the neuropathological investigation was carried out several years after an encephalitic illness and it is not possible to ascertain to what extent (if at all) the morphological changes can be attributed to the clinically documented encephalitis. Diagnosis: Severe global cerebral anoxic changes in a patient with clinically documented encephalitis.

Modern EL case 98/1133. *Royal Bath Hospital; John Radcliffe Hospital NHS Trust:* This was a 27 year old woman who was considered to have had encephalitis lethargica. The clinical details are reported by Kiley and Esiri [2].

*Pathology report:*

“Post-mortem examination revealed an active encephalitis, mainly centered on the upper brainstem and diencephalon with extensive Purkinje cell loss and marked plasma cell infiltrates and morula cells. No virus was recovered [2]”.
