# Supplementary material for: Evidence for an enterovirus as the cause of encephalitis lethargica
Source: BMC Infect Dis. 2012 Jun 20;12:136. doi: 10.1186/1471-2334-12-136 (PMC3448500; doi:10.1186/1471-2334-12-136)
Supplement: Additional file 2 — Authors’ information. [file 1471-2334-12-136-S2.doc]

**Authors’ information**

RD has a medical degree at New York University School of Medicine and spent 3 years in hospital medical practice, 3 years in general practice, and 2 years in pathology. He is now Research Fellow at the Faculty of Life Sciences, London Metropolitan University. Since 1957, RD has spent his career in the application of electron microscopy to virology and immunology. His important contribution to immunology was the discovery of the channels caused by the activation of complement on cell membranes. In virology, he has studied the attachment and entry of influenza virus particles into the epithelial cells of chick trachea. RD investigated the possibility of a virus etiology for Crohn’s disease, rheumatoid arthritis, cancer, and a number of other human diseases. With negative results, no claims for discovery were made. In addition, he has carried out electron microscopy studies in collaboration with virologists and immunologists in the investigation of the structure of immunoglobulins, the purity of virus vaccines, diagnosis of enteric viruses, and other academic and clinical applications. Summary of the number of papers published by RD: Allowing for overlaps, number of papers: 70. First authorships by RD: 21. Papers involving TEM: 60. TEM viral identification or other papers of which virology was main subject: 29. Papers on EL: 1.

SM is Director of the Clinical Pathology laboratory at the US Army Medical Research Institute in Infectious Disease (USAMRIID). He is a sub-boarded molecular pathologist and who served ten years in the Molecular Pathology Department of AFIP. He has had 20 publications, allowing for overlaps these include seven as first author, 12 in neurology, six on infectious diseases and five on EL. At AFIP, he has collaborated in a number of studies involving the molecular analysis of the genome of the “Spanish flu” influenza virus, the search for putative influenza virus as the cause of EL, medical historical studies of EL, and search for viral involvement in cases of ALS due to Guam disease.

GD is a virologist at the National Institute for Biological Standards and Control, and it is her responsibility for the verification of virus isolates and the maintenance of virus lines. She has had 6 publications on subjects related to poliovirus.

VC is a PhD student at the Faculty of Computing, London Metropolitan University, and is an expert on all applications of computing. He is currently developing new methods of computer networks.
